# Circulating ANGPTL8 as a Potential Protector of Metabolic Complications in Patients with Psoriasis

**DOI:** 10.3390/jcm12062346

**Published:** 2023-03-17

**Authors:** Anna Baran, Julita Anna Krahel, Julia Nowowiejska, Tomasz W. Kaminski, Magdalena Maciaszek, Iwona Flisiak

**Affiliations:** 1 Department of Dermatology and Venereology, Medical University of Bialystok, Zurawia 14 St., 15-540 Bialystok, Poland; 2 Pittsburgh Heart, Lung and Blood Vascular Medicine Institute, University of Pittsburgh, Pittsburgh, PA 15260, USA; 3 Department of Infectious Diseases and Hepatology, Medical University of Bialystok, Zurawia 14 St., 15-540 Bialystok, Poland

**Keywords:** psoriasis, angiopoietin-like protein 8, methotrexate, acitretin, ANGPTL8, lipid metabolism

## Abstract

Angiopoietin-like protein 8 (ANGPTL8) exerts pleiotropic effects, taking part in lipid and carbohydrate metabolism, inflammation, hematopoiesis and oncogenesis. So far, the exact molecular targets of ANGPTL8 remain poorly defined. We aimed to evaluate the serum concentration of ANGPTL8 in individuals with psoriasis and examine how systemic therapy affects the concentration of ANGPTL8. The study enrolled 35 patients with plaque-type psoriasis that were followed for 3 months of treatment with methotrexate or acitretin, and 18 healthy volunteers without psoriasis as controls. Serum ANGPTL8 concentrations were analyzed by ELISA and differences between groups were determined using Student’s *t*-test or the Mann–Whitney test, while correlations were assessed using Spearman’s rank test. The average concentration of ANGPTL8 differed significantly between the psoriasis group (before and after therapy) and the control group (*p* < 0.05). Significant negative correlations between ANGPTL8 and total cholesterol and LDL levels were noted (both *p* < 0.05). A significant increase in ANGPTL8 concentration was observed after acitretin (*p* < 0.05), whereas in patients treated with methotrexate the ANGPTL8 did not change significantly (*p* > 0.05). Additionally, a negative, statistically significant correlation with PASI was found after treatment (*p* < 0.05). Based on our study, it appears that elevated levels of ANGPTL8 may reduce the likelihood of atherogenic dyslipidemia in individuals with psoriasis, and treatment for psoriasis may impact the protective effects of ANGPTL8.

## 1. Introduction

Psoriasis is a common, chronic, stigmatizing and still incurable skin disease, affecting 2% to 4% of the world’s population [1,2]. In the past, it was considered an exclusively dermatological and rheumatic disease, affecting the skin, nails and joints. Since 2000, research into the still-unknown pathogenesis of psoriasis has exploded, which has led to the perception of psoriasis etiopathogenesis in a new light. Numerous studies have demonstrated a distinct relationship between psoriasis and the immune system as well as cardiometabolic diseases (CMDs), leading to the characterization of psoriasis as a systemic disease with an immunometabolic foundation [1,2]. Patients with psoriasis have an increased risk of developing metabolic syndrome (MS), including abdominal obesity, diabetes mellitus (DM), lipid disorders and non-alcoholic fatty liver disease (NAFLD) [3]. Individuals with psoriasis have a MS (metabolic syndrome) occurrence rate ranging from 40 to 60%, and they are twice as likely to develop MS as compared to the general population [2]. Additionally, patients with psoriasis, especially those with a severe course, are associated with higher mortality due to cardiovascular complications, such as myocardial infarction and ischemic stroke [3].

Linking this dermatosis (psoriasis) with obesity, which is a significant component of MS, is a fascinating and highly intricate task. Excessive body weight is a risk factor for psoriasis and correlates with its severity. Patients with psoriasis have a more than doubled risk of obesity. On the other hand, obesity often precedes the development of psoriatic lesions, and the BMI (body mass index) correlates with the risk of developing this dermatosis [2]. Moreover, obesity plays a key role in the development of MS, through the secretion of many pro-inflammatory cytokines and adipokines by adipose tissue, thus leading to the state of chronic inflammation, which is exacerbated in the course of psoriasis [4,5]. It causes the development of insulin resistance, lipid metabolism disorders, dysfunction of the vascular endothelium, and, consequently, atherosclerosis [6]. The progression of these interconnections is often known as the “psoriatic march,” whereby long-term inflammation in psoriasis heightens the likelihood of developing CMDs, and the constituents of MS in turn affect the severity of psoriasis [7].

So far, many factors have been identified that affect the occurrence of psoriasis and its course, such as genetic predisposition, viral and bacterial infections, environmental factors or drugs used in cardiology and immunology [1,8]. Despite that, the complex pathogenesis of psoriasis is still an enigma. Immune system disorders, in a mechanism dependent on the secretion of pro-inflammatory cytokines, and thus chronic inflammation, are still considered fundamental in the pathogenesis of the disease. Moreover, systemic inflammation has a significant impact on the etiology and development of other ailments linked with psoriasis, including atherosclerosis, obesity and other MS components, ultimately resulting in heightened mortality rates [1].

Dyslipidemia is another factor that closely connects CMDs and psoriasis [9]. Patients with psoriasis more often present a pro-atherogenic lipid profile, which consists of an increased concentration of triglycerides, low-density lipoprotein (LDL) and a decreased concentration of high-density lipoprotein (HDL) [9]. Although there is still debate over whether lipid metabolism abnormalities are the primary process in psoriasis or are a result of heightened comorbidity, dyslipidemia nonetheless plays a crucial role in the “psoriatic march” [10].

Atherosclerosis, which is a civilization disease, precedes the development of full-blown ischemic heart disease for many years. It has been proven that chronic inflammation caused by psoriasis intensifies oxidative stress, and thus accelerates the formation of atherosclerotic plaques in the blood vessel walls, which means that people suffering from psoriasis live an average of 5 years shorter, mainly due to the accelerated development of cardiovascular diseases (CVD) [10].

Psoriasis, regardless of classic risk factors, predisposes to the development of type 2 DM. The degree of risk is positively correlated with the severity of psoriasis, and individuals with both psoriasis and dyslipidemia are more prone to vascular complications than those with diabetes alone and no psoriasis [7].

One of the recently discovered proteins that play a significant role in the regulation of carbohydrate and lipid metabolism is angiopoietin-like protein 8 (ANGPTL8), also known as betatrophin, lipasin or refeeding induced fat and liver (RIFL), belonging to the ANGPTLs (angiopoietin-like proteins) family, which are structurally similar to angiopoietins but do not bind to their receptors, such as EGF-like domain 1 (Tie1) and TEK or Tie2 (endothelial-specific receptor tyrosine kinase) [11]. Angiopoietins are a family of vascular growth factors that play a key role in the angiogenesis process by regulating blood vessel permeability, vasodilation and vasoconstriction. It is noteworthy that the proteins within the ANGPTL family exhibit pleiotropic effects, contributing to the metabolism of lipids, carbohydrates, inflammation, hematopoiesis and oncogenesis. Currently, eight proteins in the ANGPTL family have been identified and are designated ANGPTL1-8 based on numerical order [12].

The gene encoding ANGPTL8 is located on chromosome 19 and is expressed mainly in the liver and adipose tissue [11]. Ren et al. showed on animal models that in mice lacking ANGPTL8 the concentration of triglycerides was reduced three times [13]. Another research group proved that the lipoprotein lipase (LPL) activity of ANGPTL8 depends on the inhibition of lipoprotein lipase [14]. Notably, ANGPTL8 is a relatively weak inhibitor of LPL compared to other members of the family, and ANGPTL 3,4,8 can interact to exert different impacts on human metabolism [15]. There are reports in the literature about the role of ANGPTL8 in the development of obesity, which is the main cause of the development of insulin resistance, and thus type 2 DM. Numerous studies have shown that the concentration of ANGPTL8 is increased in persons with obesity, MS and DM [15,16]. Another study has demonstrated that individuals with impaired glucose tolerance and type 2 diabetes mellitus exhibit notably heightened serum levels of ANGPTL8, suggesting that ANGPTL8 may play a role in the onset of this disease [17]. Taking into account the significant role of the described protein in many CMDs, it seems advisable to conduct research and broaden the knowledge about the diagnostic and clinical role of ANGPTL8 in psoriasis. A putative link between ANGPTL8 and psoriasis also appears to be the myokine irisin which has been found to increase ANGPTL8 expression during adipocyte differentiation [18]. Irisin, a thermogenic adipomyokine generated by fibronectin type III domain-containing protein 5 (FNDC5) cleavage, is implicated in adipose tissue browning and is thought to be involved in insulin resistance processes [19].

Irisin has been discovered to boost the expression of the ANGPTL8 protein during adipocyte development. Furthermore, a favorable association between the protein and irisin has been discovered in people with type 2 DM [18]. In 2017, Baran et al. proved the relationship between irisin and psoriasis and found that psoriatic patients had insignificantly higher irisin serum levels than healthy controls [19]. While there were no significant links discovered between the myokine under investigation and metabolic disorder indicators, notable positive correlations were observed between irisin and high-sensitivity C-reactive protein (hs-CRP).

Another possible link between ANGPTL8 and psoriasis might be its involvement in innate and adaptive immune responses, although the data are conflicting. There are reports on a positive correlation of circulating ANGPTL8 with hs-CRP in subjects with MS [16]. Moreover, there is an interaction with NF-κB, first reported by Zhang et al. [20]. Further, NF-κB activation is mediated by TNFα—one of the most significant cytokines in the pathogenesis of psoriasis. According to Zhang et al., ANGPTL8 functions as a negative regulator in TNFα-induced NF-κB activation, implying that the protein is a crucial step in the downregulation of inflammatory responses [20]. The circulating ANGPTL8 levels in the serum of patients with systemic inflammatory response syndrome (SIRS) were also measured by the same group and found to be significantly higher, implying that ANGPTL8 reduces the acute phase of the inflammatory response [20]. The specific involvement of ANGPTL8 in inflammatory processes and, as a result, in the progression of psoriasis is still unknown. Despite this, there exists a strong basis for future research aimed at enhancing our comprehension of the interplay between ANGPTL8 and CMS inflammation in individuals with psoriasis.

To our knowledge, despite the existence of a strong background, there is no evidence linking ANGPTL8 with psoriasis. Our aim was to investigate ANGPTL8 levels in patients with active plaque-type psoriasis and its relationship with the disease intensity, metabolic or inflammation parameters, and also define the impact of systemic therapy. Furthermore, we aimed to measure the clinical significance of ANGPTL8 in psoriasis and its potential importance in estimating the risk of CMDs in patients with psoriasis. 

## 2. Materials and Methods

The study enrolled 35 patients (13 women and 22 men) with active plaque-type psoriasis, at the median age of 49 (28–63) years, and compared them to 18 sex- and age-matched volunteers without dermatoses and family history of psoriasis. All participants signed informed consent before enrolment into the study. The inclusion criteria were as follows: age over 18 years old, diagnosis of plaque psoriasis and no systemic treatment at least 3 months prior to the study. The exclusion criteria were dietary restrictions and intake of oral medications at least 3 months prior to the study. Patients with other types of psoriasis than plaque psoriasis and suffering from chronic, autoimmune and oncological diseases were excluded from the study. Psoriasis severity was assessed utilizing the psoriasis area and severity index (PASI) and was consistently carried out by the same dermatologist. The studied group of patients was divided into three subgroups depending on the severity of the disease: PASI 1 (PASI < 10 points)—mild psoriasis, PASI 2 (PASI 10–20)—moderate psoriasis and PASI 3 (PASI > 20)—severe psoriasis. Body mass index (BMI) was calculated as weight/height^2^. In addition, all study participants were divided according to the value of the BMI: the BMI 0 subgroup was a control group, BMI 1—group of patients with normal body weight (BMI 18.5–24.9), BMI 2—overweight (BMI 25–29.9) and BMI 3—with obesity (BMI > 30). Laboratory tests, including C-reactive protein (CRP), complete blood count, serum fasting glucose, lipid profile, and indicators of liver and kidney function, were performed before and three months after the introduction of the treatment. Patients received one of two oral systemic antipsoriatic agents: 15 patients took methotrexate (MTX) 15 mg/week with folic acid supplementation (15 mg/week) and 20 patients were given acitretin, at a dose of 0.5 mg/kg/day. The study was approved by the Bioethics Committee of the Medical University of Bialystok (number: R-I-002/429/2017) and was conducted in accordance with the principles of the Helsinki Declaration [21].

### 2.1. Serum Collection

Blood samples were collected using vacuum tubes after an overnight fast. They were then allowed to clot for 30 min before centrifugation for 15 min at 2000× *g*. After centrifugation, the obtained serum was snap-frozen in liquid nitrogen and stored at −80 °C until further analysis. Laboratory parameters were measured using routine techniques. ANGPTL8 concentrations were measured with an enzyme-linked immunosorbent assay (ELISA) provided by Cloud Clone^®^, Houston, TX, USA (SEW803Hu) with a minimum detectable dose of 0.312–20 ng/mL.

Optical density was read at a wavelength of 450 nm. The concentrations were assessed by interpolation from calibration curves prepared with standard samples provided by the manufacturer. All the laboratory tests were performed by the same person in standardized laboratory settings.

### 2.2. Statistical Analysis

The values representing the normal distribution were presented as the mean (± standard deviation), while the values not satisfying the Gaussian distribution conditions were presented as the median within the 25–75 percentile of the range. The distribution of a given data set was estimated on the basis of the Shapiro–Wilk test. The differences between the two groups of variables with a parametric distribution were determined by the *p*-value on the basis of Student’s *t*-test, while the variables not meeting this condition were compared with the Mann–Whitney test. The chi-square test of independence was used to test the relationship between two nominal variables. The relationships between the examined parameters were assessed with Spearman’s rank test. The statistical significance based on *p* values at a significance level of *p* < 0.05, measuring the main effects, were considered as satisfactory to reject the null hypothesis. All calculations were performed in GraphPad 6 Prism, La Jolla, San Diego, CA, USA).

## 3. Results

The characteristics of patients and controls are listed in Table 1A,B. A total of 35 patients with active plaque-type psoriasis, 13 women and 22 men, with a median age of 49 (28–63 years) and 18 age- and sex-matched subjects as the control group, were enrolled in the study. Differences concerning BMI in controls and psoriatic patients are due to the increased cardiometabolic risk in psoriasis (Table 1A). Table 1B presents basic laboratory parameters and the psoriasis severity and activity index (PASI) in psoriatic patients.

The median value of BMI in patients was 27.6 (25.03–29.74) (Table 1A). The median basal PASI score was 17.5 (9.8–21.03) points and it decreased significantly to 10.25 (7.8–13.43) after treatment. In the study group, 9 patients had mild psoriasis (PASI < 10), 14 moderate (PASI 10–20) and 12 were diagnosed with the severe form (PASI > 20) (Table 1B). Fourteen subjects reported a positive family history of psoriasis. 

**Figure 1:**
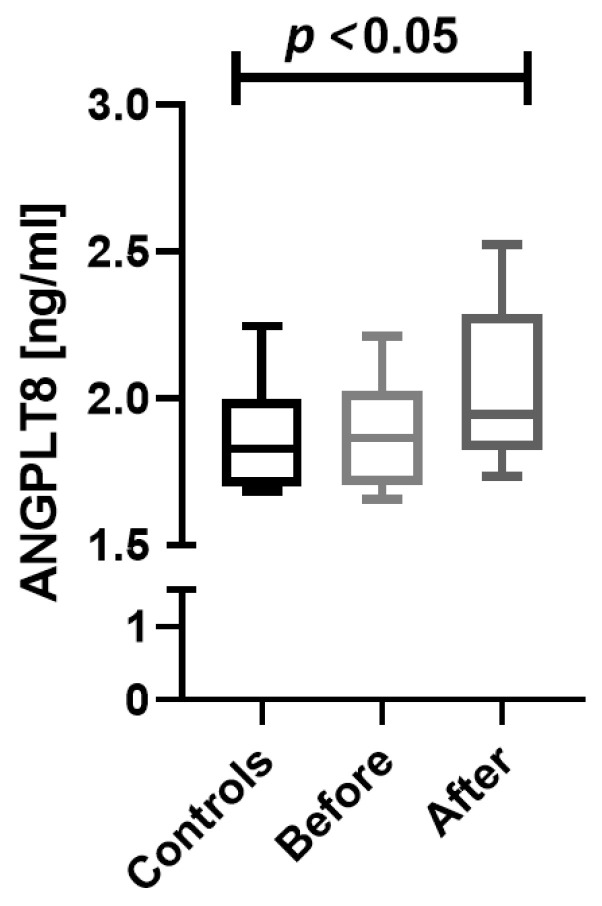
Serum ANGPTL8 concentration in controls and patients with psoriasis—before and after administered treatment.

The mean ANGPTL8 concentration was significantly different between all three groups: psoriatic patients before and after the treatment and the controls (*p* < 0.05) (Figure 1). 

ANGPTL8 did not correlate with the age of patients. ANGPTL8 was not associated with psoriasis activity expressed through the PASI score in the patients before treatment; however, there was a negative correlation between ANGPLT8 and PASI after the treatment (*p* = 0.004) (Table 2). 

**Figure 2:**
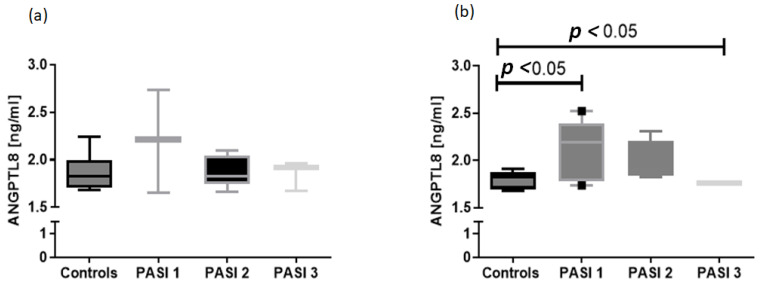
Comparison between ANGPTL8 serum concentration before (**a**) and after (**b**) treatment compared to controls, and after division into subgroups based on psoriasis severity expressed by PASI.

In addition, the median concentration of the protein was highest in patients with mild psoriasis (PASI 1) before treatment, lower in PASI 2 and the lowest in the PASI 3 subgroup (Figure 2a). The most significant increase in ANGPTL8 concentration after treatment was also observed in the PASI 3 group of patients (*p* < 0.05) (Figure 2b). 

There was no difference in ANGPTL8 concentration between the groups divided according to BMI (Table 3). ANGPTL8 did not correlate with total BMI in patients before treatment nor with CRP, RBC, WBC, glucose level or liver enzyme activity both before and after treatment (all *p* > 0.05) (Table 2). Regarding lipid metabolism indicators, ANGPTL8 significantly correlated negatively with total cholesterol and LDL (*p* = 0.029 and *p* = 0.047, respectively) (Table 2). 

After twelve weeks of systemic therapy, the skin lesions in all studied patients improved. The median of the total PASI score decreased from basal PASI 17.5 (9.8–21.03) to 10.25 (7.8–13.43) after total therapy (Table 1B). After treatment, a further increase in ANGPTL8 concentration was found in the study group (Figure 1). Additionally, a negative, statistically significant correlation with PASI was found after treatment (*p* < 0.05) (Table 2).

When analyzing the effect of the particular drug, a significant increase in ANGPTL8 concentration was observed in patients treated with acitretin (*p* < 0.05), whereas in patients treated with methotrexate, the protein level did not change significantly (*p* > 0.05) (Table 4). 

## 4. Discussion

Psoriasis is a systemic, immune-metabolic disease characterized by significant hereditary predispositions and autoimmune pathogenic mechanisms. A wide range of comorbidities emerges as psoriasis progresses, with MS playing a prominent role. Both psoriasis and CMDs share a number of pathways, most of which are related to proinflammatory pathways and cytokine profiles. It is widely known that proteins in the ANGPTL family have pleiotropic effects as a result of their participation in lipid and glucose metabolism. It is highlighted that the available information regarding the role of ANGPTL8 in human metabolism is conflicting and future studies are needed [15]. The summary of ANGPTL8 findings in regard to its role in metabolism is available in Table S1 in the Supplementary Materials. Moreover, as we mentioned earlier, ANGPTL3,4 and 8 interact together and the final impact is dependent on their cooperation. This aspect of our data makes interpretation challenging. In this paper we analyzed only ANGPTL8; however, in our earlier study, we took into consideration ANGPTL4 in psoriatic patients, but also in lichen planus and vitiligo [22]. We found out then that there was no significant difference in serum ANGPTL4 between psoriatic patients and controls [22], which provides an additional piece of information to the current understanding of the ANGPTL protein family in the context of psoriasis.

There is evidence of elevated levels of ANGPTL8 observed in individuals diagnosed with CMDs, and this association is reported to be significantly correlated with hs-CRP levels, underscoring its potential involvement in the metabolic and inflammatory pathways that play an important role in the advancement of psoriasis [16]. On the other hand, there are reports, albeit fewer compared to other findings, on higher concentrations of ANGPTL8 in subjects with CAD in relation to their better survival due to fewer cardiovascular incidents [15]. This may suggest the multifaceted nature of ANGPTL8. Therefore, we intended to explore the potential interplay between ANGPTL8 and the complexity of psoriasis. In the present study, we found that serum ANGPTL8 concentration was significantly different in psoriatic patients before and after the treatment and compared to controls. To the best of our knowledge, there is only one other paper investigating ANGPTL8 in psoriasis. Holmannova et al. in 2020 examined 44 psoriasis patients (22 without MS and 20 with MS) and 80 controls (44 controls without MS and 36 controls with MS). There were no significant variations in ANGPTL8 serum levels between patients and controls, even within subgroups. When compared to those without MS, the presence of MS has no effect on the level of ANGPTL8 in either the patient or control group [23]. These inconsistencies may be clarified by the complexity of ANGPTL8, its participation in various processes or the techniques used for ANGPTL8 detection. Some commercial kits only detect the full-length version of ANGPTL8, whereas others test both the full-length and cleaved C- and N-termini. The authors hypothesized as to why, despite the presence of inflammation and MS, the level of ANGPTL8 did not rise as expected. The authors suggest that despite the increasing knowledge about ANGPTL8, there are still many unknown factors and methods that could affect its production and function. These factors include inhibitors or modifiers of ANGPTL8 synthesis, interactions of ANGPTL8 with other molecules, the role of microbiota in regulating lipid metabolism and immune system function, and the presence of truncated variants of ANGPTL8 [23]. According to our data, we believe ANGPTL8 may be a protective agent in a psoriatic environment, similar to what is observed in CAD. The strong negative correlations between ANGPTL8 and total cholesterol and LDL may point to a protective role of the protein in terms of atherogenic dyslipidemia development. 

The highest concentration of ANGPTL8 was found among patients with mild psoriasis and a lower concentration in the other two groups with more severe skin lesions. This observation could indicate that in subjects with mild psoriasis, the impact of ANGPTL8 is the most significant. 

There is no additional information about the role of ANGPTL8 under psoriatics conditions in current literature data. ANGPTL8 has been shown to have a critical role in different systemic disorders, such as metabolic disorders, cardiovascular diseases and cancer. However, the data on ANGPTL8 are still inconsistent and further research is required to better understand its role in these different areas of medicine. The discovery that ANGPTL8 could be a hormone produced by the liver and capable of promoting cell proliferation, hence the name betatrophin [24], piqued interest in the protein. Yi et al. reported that injecting naked DNA into the tail vein of mice to overexpress ANGPTL8/betatrophin resulted in a 17-fold increase in β cell replication, with betatrophin administration resulting in a 3-fold increase in β cell area [24]. As a result, two later investigations [25,26] found that mice lacking ANGPTL8 have normal glucose levels and β cell mass when fed a high-fat diet. Overexpression of ANGPTL8 in mice did not result in increased proliferation of β cells [26]. Recombinant ANGPTL8 was also shown to exert no stimulation on β cell proliferation in a collaborative investigation combining three different laboratories [27]. The publication by Yi et al. [24] on the hypothesized betatrophin function of ANGPTL8 was retracted as a result of these persuasive data. Moreover, insulin upregulates ANGPTL8 gene expression in human adipose tissue in vivo, according to biopsies from participants who underwent hyperinsulinemic–euglycemic clamp [28]. Furthermore, Zhang et al. found that insulin alone raises ANGPTL8 gene expression in cultured mouse hepatocytes and mature adipocytes in a dose-dependent manner, but high glucose coupled with insulin promotes further upregulation of ANGPTL8 expression solely in adipocytes [29]. As a result, it has been proposed that the favorable effects of insulin and glucose on ANGPTL8 expression in the liver and adipose tissue are due to different mechanisms [29]. Insulin appears to boost ANGPTL8 expression in the adipose and liver, but whether it affects ANGPTL8 release into the circulation requires more research. DM type 2, atherosclerosis and non-alcoholic steatosis have all been linked to increased circulating levels of ANGPTL8 [30,31,32,33]. Furthermore, plasma levels of ANGPTL8 have been found to be higher in patients with severe infections, and a strong association has been observed in animal models between circulating ANGPTL8 and the LPS-induced acute inflammatory response [20]. The involvement of ANGPTL8 in controlling blood lipoproteins, combined with these findings, suggests that ANGPTL8 may play a role in inflammation. Notably, there was no correlation between ANGPTL8 and CRP in our study, implying that higher ANGPTL8 levels in psoriatic patients are not associated with the severity of inflammation and, furthermore, with the extent of the disease. This new hypothesis illuminates the actual diagnostic and therapeutic worth of this protein, revealing that it is an unreliable indicator of inflammatory conditions in psoriasis. Plasma levels of ANGPTL8 are known to be higher in atherosclerotic patients, resulting in plasma LPL inhibition and the build-up of circulating lipoproteins, both of which contribute to inflammation and the pathogenesis of atherosclerosis. 

NAFLD is rapidly becoming a well-known condition linked to obesity, insulin resistance, type 2 DM, cardiovascular diseases and also psoriasis [5,34]. NAFLD progresses to non-alcoholic steatohepatitis (NASH) in many individuals, eventually becoming a significant risk factor for hepatocellular cancer [5,33]. Increased blood ANGPTL8 levels have been linked to the severity of NAFLD in recently published investigations [35,36]. ANGPTL8 expression in the liver has also been observed to be higher in multiple mouse models of fatty liver, suggesting that ANGPTL8 could be a potential biomarker for NAFLD [32]. Various types of stress, such as oxidative stress or dietary excess that contributes to hyperglycemia and hypertriglyceridemia, which are well-documented risk factors for the pathogenesis of NAFLD [37], also lead to enhanced hepatic ANGPTL8 production and secretion [38], as mentioned above. The exact cause-and-effect relationship between increased ANGPTL8 and NAFLD pathogenesis, however, remains unknown but it would be interesting to investigate in psoriatic subjects. As for liver disorders, in our study we did not find any significant correlation between ANGPTL8 and liver enzyme activity.

After the treatment, the ANGPTL8 concentration in our patients increased, and regarding the particular systemic agent, this increase was noted only in subjects treated with acitretin. Unfortunately, we were unable to find any data on the possible influence of antipsoriatic agents on ANGPTL8. Nonetheless, our study indicates that antipsoriatic treatment may modify the protective characteristics of ANGPTL8, considering that it was elevated following the therapy.

Perhaps considering ANGPTL8 is lower before the treatment and increases after its administration and is additionally correlated with PASI after therapy, may mean that its beneficial role in psoriasis is diminished under the condition of active dermatosis, whereas it is restored by properly administered treatment. Nevertheless, future studies on a larger number of enrolled patients are required to find out the role of ANGPTL8 in such a complex disease as psoriasis.

Our study has a limitation in that we only enrolled a small number of participants from a single ethnicity. Additionally, we focused on analyzing only one marker, ANGPTL8, while it would be beneficial to investigate it in conjunction with ANGPTL3 and 4 in a single study, as they mutually influence each other. The period of follow-up is also not long enough, and we would like to extend the time of observation in the future.

## 5. Conclusions

We investigated ANGPTL8 in psoriatic patients with regard to clinical and laboratory data, as well as the administered treatment in order to reveal its presumable role in this specific group of patients. We suspect ANGPTL8 to act as a potentially protective agent in psoriasis in terms of cardiometabolic complications development. In subjects with mild psoriasis, the impact of ANGPTL8 seems most significant. High concentrations of ANGPTL8 may decrease the risk of atherogenic dyslipidemia in psoriatics. Antipsoriatic treatment may modulate the protective properties of ANGPTL8.

## Figures and Tables

**Table 1:** (A) Basic characteristics of patients with psoriasis and controls without psoriasis. (B) Basic characteristics of the patient group with treatment.

(A)		
Parameter	Controls (*n* = 18)	Patients (*n* = 35)
Sex (M/F)	9/9	22/13 NS
Age (years)	30 (28–57.25)	49 (28–63) NS
Height (cm)	176.5 (164–180.5)	176 (166–180)
Weight (kg)	72.5 (59.5–83.5)	83 (74–90) *
BMI	23.1 (21.1–25.48)	27.6 (25.03–29.74) **
**(B)**		
**Characteristics**		**Values**
PASI before treatment		17.5 (9.8–21.03)
PASI after treatment		10.25 (7.8–13.43) ***
RBC (×10^3^/mL)		4.58 ± 0.09
PLT (×10^3^/mL)		228 ± 11.38
WBC (×10^3^/mL)		7.52 (6.25–8.42)
Glucose (mg/dL)		82 (79–98.5)
Total cholesterol (mg/dL)		168.8 ± 4.99
Triglycerides (mmol/L)		121 ± 8.44
HDL (mmol/L)		47.1 ± 2.09
LDL (mmol/L)		102.6 ± 4.2
CRP (mg/L)		2.88 (1–7.72)
ALT (U/L)		16.5 (11.75–28)
AST (U/L)		19.25 (15.75–30.5)

*/**—statistical significance with *p* values < 0.05/<0.01, respectively, between controls and patients. ***—statistical significance with *p* values < 0.001 after treatment compared to the values before treatment. RBC, red blood cells; PLT, platelets; WBC, white blood cells; HDL, high-density lipoproteins; LDL, low-density lipoproteins; CRP, C-reactive protein; ALT, alanine aminotransferase; AST, asparagine aminotransferase; PASI, psoriasis activity and severity index.

**Table 2:** The presence of correlations between ANGPTL8 serum concentrations and studied parameters before and after treatment in psoriatic patients.

Characteristics	Before Treatment	After Treatment
Sex (M/F)	0.065	−0.094
Age (years)	0.361	−0.005
BMI	−0.143	0.005
PASI before treatment	−0.187	-
PASI after treatment	-	−0.628 (0.004) **
RBC (×10^3^/mL)	−0.446	0.158
PLT (×10^3^/mL)	−0.156	*−0.444* (*0.057*)
WBC (×10^3^/mL)	−0.156	−0.025
Glucose (mg/dL)	0.170	−0.011
Total cholesterol (mg/dL)	−0.585 (0.029) *	−0.096
TGs (mmol/L)	−0.37	0.093
HDL (mmol/L)	−0.291	−0.014
LDL (mmol/L)	−0.48 (0.047) *	−0.124
CRP (mg/L)	0.046	−0.172
ALT (U/L)	0.126	0.037
AST (U/L)	0.066	0.144

*/** indicates statistical significance with *p* values < 0.05 and <0.01. *Italic* font indicates a trend. RBC, red blood cells; PLT, platelets; WBC, white blood cells; HDL, high-density lipoproteins; LDL, low-density lipoproteins; CRP, C-reactive protein; ALT, alanine aminotransferase; AST, asparagine aminotransferase; PASI, psoriasis activity and severity index; BMI, body mass index.

**Table 3:** Comparison between ANGPTL8 serum concentrations before and after treatment in subgroups of psoriatic patients divided according to BMI, and in controls without psoriasis.

ANGPTL8	Controls	BMI 1	BMI 2	BMI 3
Before	1.83 (1.68–2.25)	1.97 (1.74–2.07)	1.71 (1.66–2.21)	1.87 (1.66–2.74)
After	-	2 (1.74–2.51)	2.15 (1.76–2.36)	1.87 (1.77–2.52)

**Table 4:** Comparison between ANGPTL8 serum concentrations before and after treatment with acitretin and methotrexate separately in psoriatic patients, and in controls without psoriasis.

ANGPTL8	Controls	Acitretin	Methotrexate
Before	1.83 (1.68–2.25)	1.86 (1.66–2.74)	1.89 (1.66–2.1)
After	-	2.16 (1.76–2.52) *	1.84 (1.74–2.29)

*—statistical significance with *p* < 0.05, compared to controls. ANGPTL8 levels were highest in patients with normal body weight, but not statistically significant.

## Data Availability

The data are available upon request from the corresponding author.

## Supplementary Materials

The following supporting information can be downloaded at: https://www.mdpi.com/article/10.3390/jcm12062346/s1, Table S1: Summary of original studies regarding the role of ANGPTL8 in metabolism.
