# Supplementary material for: Circulating ANGPTL8 as a Potential Protector of Metabolic Complications in Patients with Psoriasis
Source: J Clin Med. 2023 Mar 17;12(6):2346. doi: 10.3390/jcm12062346 (PMC10058172; doi:10.3390/jcm12062346)
Supplement: Supplementary file 1 [file jcm-12-02346-s001.zip › jcm-2206835-supplementary.pdf]

Table S1. Summary of original studies regarding the role of ANGPTL8 in metabolism.

Study	Main Findings about ANGPTL8	Population
Ren et al. 2012	regulator of lipid metabolism of prolipogenic function; upregulation of ANGPTL8 in adipose tissue and liver in genetic obesity and by nutritional refeeding; ANGPTL8 transcript expression level in adipocytes decreased by agents stimulating lipolysis	mice
Zhang 2012	regulator of lipid metabolism of prolipogenic function; obesity increases liver ANGPTL8, whereas fasting reduces its expression in fat; ANGPTL8 overexpression by adenoviruses in mice increases serum triglycerides; recombinant ANGPTL8 inhibits LPL activity	mice
Wang et al. 2013	mice lacking ANGPTL8 present improper triglyceride metabolism without impaired glucose metabolism; mice lacking ANGPTL8 present lower plasma concentration of triglycerides	mice
Gusarova et al. 2014	deletion of ANGPTL8 leads to reduction in plasma triglyceride concentrations; ANGPTL8 overexpression leads to elevation of plasma triglyceride concentrations; ANGPTL8 does not affect glucose metabolism	mice
Nidhina Haridas et al. 2015	circulating ANGPTL8 concentration do not reflect insulin action in liver or adipose tissue	humans
Yamada et al. 2015	circulating concentrations of ANGPTL8 are elevated in Japanese with diabetes mellitus; log ANGPTL8 correlates positively with HbA1c and log triglycerides, negatively with HDL-C	humans
Cox et al. 2016	ANGPTL8 administration may stimulate dyslipidemia	mice
Lee et al. 2016	circulating ANGPTL8 correlates positively with obesity, glycemic indices, liver enzymes, and was increased in mice and humans with NAFLD	humans, mice, cells
Abu-Farha et al. 2016	potential predictive marker of MS; ANGPTL8 level higher in subjects with MS compared to controls; strong positive correlation between ANGPTL8 and hsCRP	humans
Mele et al. 2017	ANGPTL8 concentration significantly lower in patients with Prader-Willy syndrome than match controls; ANGPTL8 correlates with liver enzymes activity and severity of liver steatosis	humans
Yin et al. 2017	serum ANGPTL8 concentrations significantly increased in subjects with impaired glucose tolerance and type 2 DM; ANGPTL8 might be involved in hyperglycemia;	humans
Zhang et al. 2017	pro-inflammatory cytokines stimulate ANGPTL8; ANGPTL8 inhibits NF-KB	humans, mice, cells
von Loeffelholz, 2017	circulating ANGPTL8 correlates with liver steatosis, triglycerides, saturated, monounsaturated and polyunsaturated fatty acids; weight loss results in reduction of ANGPTL8 concentration	humans
Zhang et al. 2019	regulator of lipid metabolism of prolipogenic function; ANGPTL8-lacking mice have improved glucose tolerance and reduced fed and fasted plasma triglyceride concentration; increased ANGPTL8 mRNA expression after stimulation with insulin in liver and adipose tissue	mice, cells
Holmannova et al. 2020	no significant differences in the serum concentration of ANGPTL8 between psoriatics and controls; MS diagnosis did not affect ANGPTL8 levels	humans

ANGPTL8, angiopoietin-like protein 8; MS, metabolic syndrome, NAFLD, non-alcoholic fatty liver disease; hs-CRP, high-sensitivity C-reactive protein; DM, diabetes mellitus; HDL, high-density lipoprotein; log, logarithm; LPL, lipoprotein lipase.
